# C-Reactive Protein as a Risk Marker for Post-Infarct Heart Failure over a Multi-Year Period

**DOI:** 10.3390/ijms22063169

**Published:** 2021-03-20

**Authors:** Iwona Świątkiewicz, Przemysław Magielski, Jacek Kubica

**Affiliations:** 1 Department of Cardiology and Internal Medicine, Nicolaus Copernicus University in Toruń, Collegium Medicum in Bydgoszcz, 85-094 Bydgoszcz, Poland; przemyslaw.magielski@wp.pl (P.M.); jwkubica@gmail.com (J.K.); 2 Division of Cardiovascular Medicine, University of California San Diego, La Jolla, CA 92037, USA

**Keywords:** heart failure, acute myocardial infarction, inflammation, biomarkers, C-reactive protein, ischemic heart disease, echocardiography, remodeling

## Abstract

Inflammatory activation during acute ST-elevation myocardial infarction (STEMI) can contribute to post-infarct heart failure (HF). This study aimed to determine prognostic value of high-sensitivity C-reactive protein concentration (CRP) for HF over a long-term follow-up in 204 patients with a first STEMI undergoing guideline-based therapies including percutaneous coronary intervention. CRP was measured at admission, 24 h (CRP_24_), discharge (CRP_DC_), and one month (CRP_1M_) after index hospitalization for STEMI. Within a median period of 5.6 years post-index hospitalization for STEMI, hospitalization for HF (HFH) which is a primary endpoint, occurred in 24 patients (11.8%, HF+ group). During the study, 8.3% of HF+ patients died vs. 1.7% of patients without HFH (HF- group) (*p* = 0.047). CRP_24_, CRP_DC_, and CRP_1M_ were significantly higher in HF+ compared to HF- group. The median CRP_1M_ in HF+ group was 2.57 mg/L indicating low-grade systemic inflammation, in contrast to 1.54 mg/L in HF- group. CRP_1M_ ≥ 2 mg/L occurred in 58.3% of HF+ vs. 42.8% of HF- group (*p* = 0.01). Kaplan–Meier analysis showed decreased probability of survival free from HFH in patients with CRP_24_ (*p* < 0.001), CRP_DC_ (*p* < 0.001), and CRP_1M_ (*p* = 0.03) in quartile IV compared to lower quartiles. In multivariable analysis, CRP_DC_ significantly improved prediction of HFH over a multi-year period post-STEMI. Persistent elevation in CRP post STEMI aids in risk stratification for long-term HF and suggests that ongoing cardiac and low-grade systemic inflammation promote HF development despite guideline-based therapies.

## 1. Introduction

Heart failure (HF) remains a significant complication post myocardial infarction (MI) and is associated with high cardiovascular mortality [1,2,3,4]. Despite guideline-based therapies which lead to decreased mortality of patients with acute ST-segment elevation myocardial infarction (STEMI), HF occurs in up to 30% of STEMI patients depending on the time point of assessment, diagnostic criteria for HF, and therapeutic approach for STEMI [1,2,3,4,5]. Hospital readmission for HF, especially repeated hospitalizations, are associated with an adverse outcome [6,7,8]. Strategies to reduce post-infarct HF and readmissions for HF are challenging yet critical for improving long-term outcome. The strategies include early identification of high-risk patients for addressing causes of HF and optimizing evidence-based therapies.

Inflammatory activation during acute MI is associated with both myocardial damage and repair. However, enhanced inflammatory response leads to further cardiac injury and progressive left ventricular (LV) dysfunction and remodeling (LVR) which can contribute to HF development [5,9,10,11,12,13]. Cardiac inflammation in response to MI may also trigger systemic inflammation that accelerates atherosclerosis progression, endothelial dysfunction, and vascular remodeling. This can promote HF development [9,14,15,16]. Cardiac release of various cytokines such as interleukin (IL)-1 family and IL-6 was documented in patients with acute STEMI [13,17,18,19]. Higher IL-6 was associated with increased risk of post-infarct HF independently of LV ejection fraction (LVEF) and management strategy [18,20].

Inflammation plays significant role in the pathophysiology of HF [21,22]. Response to myocardial injury involves activation of various inflammatory pathways and increased production of pro-inflammatory cytokines such as IL-1 and IL-6 in the peripheral circulation and heart. In patients with HF, the level of pro-inflammatory cytokines increases as HF worsens and is associated with diminished exercise capacity and increased risk of death and hospitalization for HF (HFH) [23,24,25,26]. Expression of various cytokines leads to alterations in myocyte contractility, cardiac remodeling and fibrosis, LV dysfunction and LVR, a low-grade systemic inflammation, and endothelial dysfunction, which can serve as predictors of cardiovascular events, including HFH [13,15,16,21,22,23,24,27,28,29,30,31].

C-reactive protein (CRP) concentration reflects inflammation and serves as a downstream biomarker for IL-6 and IL-1 activity [32]. At present, CRP is the preferred biomarker of inflammation for cardiovascular risk stratification [33,34]. Previous studies on CRP as a risk marker for STEMI patients were mostly focused on clinical endpoints other than HF and had several methodological limitations such as a lack of treatment with percutaneous coronary intervention (PCI) and evidence-based pharmacotherapies in all patients, CRP measurement at a single time point, no use of high-sensitivity CRP assays, and/or short-term follow-up (e.g., [12,18,19,35,36,37,38,39,40,41]). A few studies which examined prognostic value of CRP for HF post-STEMI did not elucidate the question of usefulness of CRP measurement for post-infarct HF over a long-term period [18,19,39]; however, these studies had limitations associated with the use of composite endpoints and guideline-based therapies only in portion of patients, and lacked multiple CRP measurements at various time-points of acute STEMI as well as long-term follow-up. Although van Diepen et al. [18] showed that elevated baseline CRP predicts composite of all-cause death, shock or HF in STEMI patients, the study involved a short-term follow-up of 90 days. Prognostic significance of CRP for HF and its outcome was demonstrated in various clinical settings [25,37,42,43,44,45]; however, data on CRP as a risk marker for HF over a long-term follow-up in STEMI patients undergoing guideline-based therapies remain limited.

In our previous studies of patients with acute STEMI treated with PCI we found that elevated CRP predicts LV systolic dysfunction (LVSD) and LVR, and points to an increased risk of HF in patients with post-infarct LVSD [5,10,11]. The present study extends the previous work and aims to determine the prognostic value of high-sensitivity CRP concentration during acute STEMI for the risk of HF in long-term follow-up in a homogenous population of patients with a first STEMI undergoing primary PCI and guideline-based therapies. Hospitalization for HF (HFH) in the long-term follow-up is considered as primary study endpoint and is defined as post-index STEMI readmission due to new or increasing symptoms and signs of HF in combination with a change in treatment to improve HF. This primary endpoint has been selected because the HFH validates the HF diagnosis and is associated with subsequent increase in risk of mortality [7]. All-cause death in long-term follow-up and an incidence of CRP_1M_ ≥ 2 mg/L at one-month post-index hospitalization for STEMI are considered as secondary study endpoints. The protocol of this study includes rigorous selection criteria of patients, multi-year follow-up, and multiple time-points for collecting data including an incidence of major adverse cardiac events (MACE). Serial multiple measurements of high-sensitivity CRP concentration were made to determine the optimal time point that is the strongest predictor of primary endpoint.

## 2. Results

Throughout the study period, 4311 patients with acute MI were hospitalized for PCI in our center including 2730 patients with acute STEMI who were treated with primary PCI. These patients were screened to determine their eligibility for this study. In total, consecutive 204 eligible STEMI patients (156 men and 48 women) were enrolled in this study and subject to long-term follow-up with median observation period of ~5.6 years (4.9–6.3 years). The enrolled patients satisfied inclusion criteria and gave informed consent. Detailed description of study design, eligibility criteria, methodology, and therapy is provided in Section 4. Additional details about the patient selection process is presented elsewhere [5]. All enrolled patients received standard guideline-based treatment including primary PCI following current guidelines on management of acute STEMI as described in Section 4.

Within a median period of 5.6 years, total MACE as defined in Section 4, occurred in 89 patients (43.6% of total). During the entire study, 5 patients (2.5% of total) died. Non-fatal MI, ischemic stroke, and hospitalization for unstable angina leading to revascularization occurred in 29 patients (14.2% of total), 3 patients (1.5%), and 59 patients (28.9%), respectively. In total, there were 5 deaths, 30 non-fatal MI (1 patient had 2 events), 4 ischemic strokes (1 patient had 2 events), and 66 hospitalizations for unstable angina leading to revascularization (7 patients had 2 events).

### 2.1. Study Endpoints

#### 2.1.1. Hospitalization for Heart Failure and All-Cause Death

Over a median period of 5.6 years post-index hospitalization for STEMI, HFH (i.e., a primary study endpoint) occurred in 24 patients (11.8% of total). The patients with HFH are denoted as HF+ group. In total, there were 37 HFH during entire study period as some patients were hospitalized more than once. Specifically, 11 patients (45.8% of HF+ group) had one HFH and 13 patients (54.2% of HF+ group) had ≥ 2 readmissions for HF worsening, including 6 patients who were hospitalized for HF 3–6 times. The median time between the hospital discharge upon index hospitalization for STEMI and the first hospital readmission for HF symptoms was 210 days (38–703 days).

Over a long-term follow-up, total MACE was more prevalent in HF+ group than HF- group (83.3% of patients vs. 42.2%, *p* < 0.001). Specifically, all-cause death (i.e., a secondary study endpoint) occurred more frequently in patients requiring HFH compared to patients without HFH (2 patients, 8.3% of HF+ group vs. 3 patients, 1.7% of HF- group, *p* = 0.047). Note that the patients without HFH are denoted as HF- group. Patients from HF+ group were hospitalized more frequently for unstable angina leading to revascularization compared to HF- group (58.3% of patients vs. 25%, *p* < 0.001). No significant differences in the incidence of non-fatal MI and ischemic stroke were observed between HF+ and HF- groups (12.5% of patients vs. 14.4%, *p* = 0.798 and 4.2% vs. 1.1%, *p* = 0.243, respectively).

The comparison of baseline characteristics during index hospitalization for STEMI for the groups of patients with and without HFH in long-term follow-up is given in Table 1.

Patients requiring HFH in long-term follow-up had higher body mass index (BMI) and longer STEMI-induced pain, as well as more frequent anterior STEMI, diabetes mellitus (DM), and HF symptoms at hospital discharge upon index STEMI than patients without HFH. Patients from HF+ group had higher myocardial necrosis indices and B-type natriuretic peptide (BNP) levels during index hospitalization for STEMI compared to HF- group. No differences in age, sex, prevalence of hypertension and HF prior to STEMI, as well as other biochemical parameters were observed between groups. Also, no differences in angiographic indices during primary PCI for index STEMI were found between groups except for more frequent infarct-related left descending artery and abciximab use in HF+ compared to HF- group. Most patients in both groups had multivessel coronary artery disease. In the majority of patients (up to 96%) in both groups, PCI resulted in reperfusion success, more often in HF+ group. All patients from HF+ group and almost all HF- patients received at discharge upon index hospitalization for STEMI and continued for at least 12 months beta blocker, angiotensin-converting enzyme inhibitor, and high-intensity statin therapy. No differences in the use of aspirin and other evidence-based pharmacotherapy were observed between groups except for diuretics which were prescribed more frequently in HF+ than HF- group.

Echocardiography at discharge upon index hospitalization for STEMI revealed larger LV volumes and LV mass, and more impaired LV function, both systolic and diastolic, in patients requiring HFH in long-term follow-up compared to those without HFH (Table 2). After six months, further deterioration of LV morphology and function was observed in HF+ group (Table 2). LVR at six months after index STEMI (LVR_6M_) was more frequent in patients from HF+ than HF- group (58.3% of patients vs. 23.6%, *p* < 0.001). The incidence of LVSD at discharge upon index STEMI (LVSD_DC_) and LVSD six months after index STEMI (LVSD_6M_) was higher in HF+ than HF- group (70.8% of patients vs. 22.2%, *p* < 0.001 and 66.7% vs. 10.9%, *p* < 0.001, respectively).

**Figure 1:**
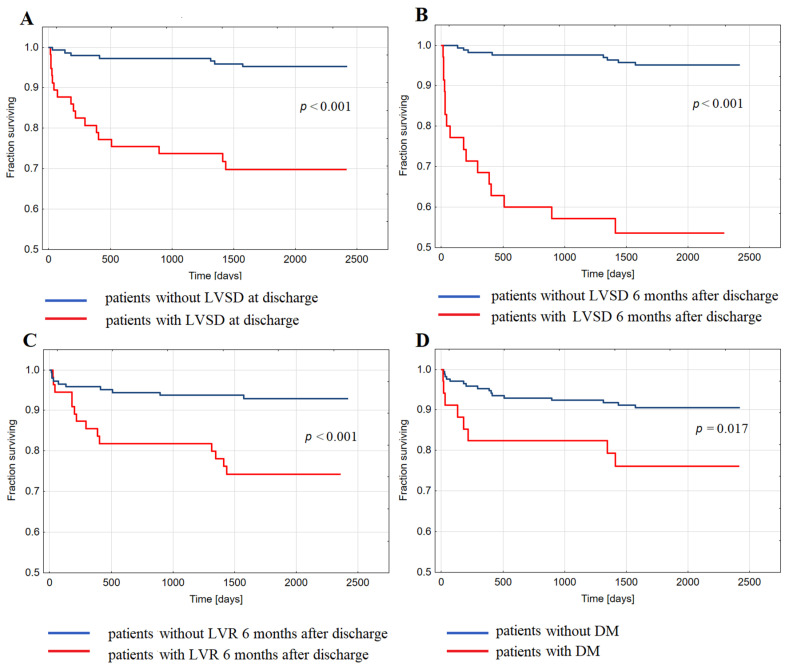
Kaplan–Meier analysis showing survival free from the hospitalization for heart failure in long-term follow-up after discharge upon index ST-elevation myocardial infarction in groups of patients: (**A**) with and without left ventricular systolic dysfunction (LVSD) at discharge; (**B**) with and without LVSD six months after discharge; (**C**) with and without left ventricular remodeling (LVR) six months after discharge; (**D**) with and without diabetes (DM).

HFH in long-term follow-up occurred more often in patients with LVSD_DC_ than without LVSD_DC_ (29.8% of patients vs. 4.8%, *p* < 0.001), in patients with LVSD_6M_ than without LVSD_6M_ (45.7% vs. 4.9%, *p* < 0.001), and in patients with LVR_6M_ than without LVR_6M_ (25.5% vs. 7%, *p* < 0.001). Also, HFH was more prevalent in patients with DM than without DM (24.3% of patients vs. 9.0%, *p* = 0.009). Kaplan–Meier analysis showed decreased probability of long-term survival free from HFH post-index hospitalization for STEMI in patients with LVSD_DC_ (Figure 1A), LVSD_6M_ (Figure 1B), LVR_6M_ (Figure 1C), and DM (Figure 1D) compared to patients without LVSD, LVR_6M_, and DM, respectively.

#### 2.1.2. C-Reactive Protein

**Figure 2:**
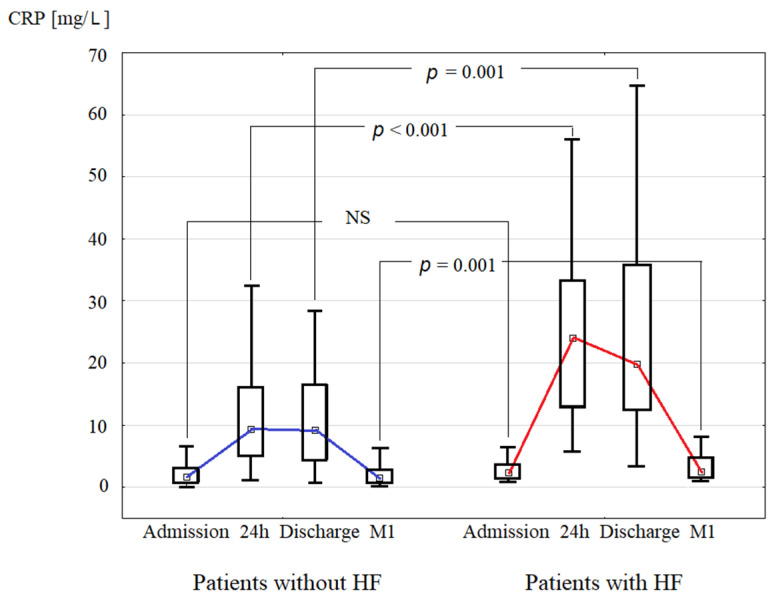
Comparison of C-reactive protein (CRP) median concentration during index hospitalization for ST-elevation myocardial infarction (at admission, 24 h, and discharge), and at one month after discharge (M1) between groups of patients without and with hospitalization for heart failure (HF) in long-term follow-up.

CRP concentration was measured at admission (CRP_AD_), 24 h after admission (CRP_24_), at discharge (CRP_DC_), and one month after discharge (CRP_1M_) upon an index hospitalization for acute STEMI. CRP concentration increased during the first 24 h and was elevated at discharge in the HF+ patients to a greater extent than in the HF- patients, and hence CRP_24_ and CRP_DC_ were significantly higher in HF+ compared to HF- group (Table 1, Figure 2). Also, patients from HF+ group had elevated CRP_1M_ (median = 2.57 mg/L), in contrast to significantly lower median CRP_1M_ (1.54 mg/L) in HF- group (Table 1). The incidence of CRP_1M_ ≥2 mg/L (i.e., a secondary study endpoint) was 44.6% in the whole study population. CRP_1M_ ≥ 2 mg/L was more prevalent in HF+ compared to HF- group (58.3% vs. 42.8%, *p* = 0.01).

**Figure 3:**
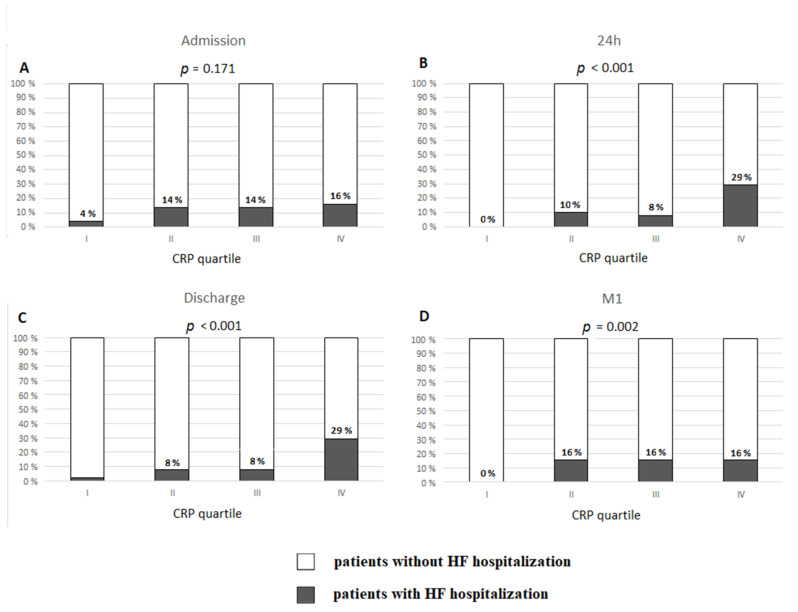
Occurrence of hospitalization for heart failure in long-term follow-up post-discharge upon index hospitalization for ST-elevation myocardial infarction based on quartiles of C-reactive protein (CRP) concentration: (**A**) at admission; (**B**) 24 h after admission; (**C**) at discharge; (**D**) one month after discharge (M1).

The analysis of CRP levels by quartiles revealed that higher CRP_24_, CRP_DC_, and CRP_1M_ were associated with higher incidence of HFH (Figure 3). HFH occurred in 29.4% of patients with CRP_24_ in the fourth quartile (cut-off value ≥ 19.64 mg/L) vs. 0% in the first quartile (≤5.62 mg/L) (*p* < 0.0001), and in 29.4% of patients with CRP_DC_ in the fourth quartile (≥17.65 mg/L) vs. 2% in the first quartile (≤4.91 mg/L) (*p* < 0.0001). No patients from HF+ group had CRP_1M_ in the first quartile (cut off value < 0.87 mg/L). However, 16% of patients who had the values of CRP_1M_ in each of the remaining quartiles required HFH.

**Figure 4:**
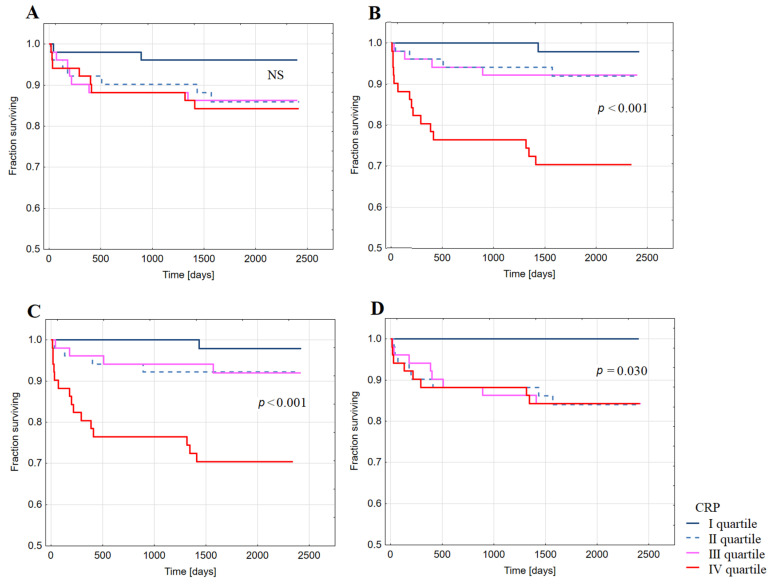
Kaplan–Meier analysis showing survival free from the hospitalization for heart failure in long-term follow-up post-discharge upon index hospitalization for ST-elevation myocardial infarction in groups of patients with CRP concentration in the quartiles of: (**A**) CRP at admission; (**B**) CRP 24 h after admission (CRP 24 h); (**C**) CRP at discharge; (**D**) CRP at one month after discharge (CRP M1).

Kaplan–Meier analysis showed decreased probability of long-term survival free from HFH after index hospitalization for STEMI in patients with CRP in the highest quartiles of CRP_24_ (Figure 4B), CRP_DC_ (Figure 4C), and CRP_1M_ (Figure 4D). No significant difference in survival free from HFH was observed between groups of patients with CRP in different quartiles of CRP_AD_ (Figure 4A).

### 2.2. Predictors of Hospitalization for Heart Failure

Univariate logistic regression analysis revealed predictors of HFH in long-term follow-up after index hospitalization for STEMI (Table 3). The univariate analysis includes parameters from Table 1 and 2 with a *p*-value < 0.01, as well as CRP and leukocyte count during index hospitalization for STEMI regardless of *p*-value.

In the multivariable logistic regression model, low LVEF and an average peak systolic mitral annular velocity (S’) at discharge, and high creatinine kinase myocardial band (CK-MB) at admission during index hospitalization for STEMI identified increased risk of HFH in long-term follow-up (Table 3). The second multivariable model that excluded LVEF at discharge upon index STEMI found high wall motion score index (WMSI) at discharge, BMI at admission, longer duration of STEMI-induced pain, and high CRP_DC_ upon index hospitalization for STEMI as independent predictors of HFH in long term follow-up post-index hospitalization for STEMI (Table 3).

## 3. Discussion

The findings of this study support the value of CRP as a risk marker for post-infarct HF over a multi-year period. This study provides evidence that persistent elevation in CRP is associated with increased risk of hospitalization for HF (HFH) and HF-related mortality in long-term (~6-year) follow-up for a population with acute STEMI receiving early successful PCI and guideline-based therapies. Elevated CRP during an index hospitalization for STEMI and one month upon discharge indicates ongoing cardiac and low-grade systemic inflammation, which can contribute to the development of post-infarct HF. Our findings indicate a significance of inflammation-related mechanisms which are associated with the initiation and progression of post-infarct HF. Our results provide further support for targeting inflammation to reduce morbidity and mortality related to post-infarct HF that is highly prevalent and is associated with adverse outcome despite evidence-based treatment. A unique attribute of our study is the focus on investigating the value of high-sensitivity CRP concentration during acute STEMI for predicting the risk of HF in a multi-year period performed on homogenous population of patients with a first STEMI undergoing prompt reperfusion and early neurohormonal blockade.

The primary findings of our study are: (i) ~12% of patients with first STEMI undergoing early successful PCI and guideline-based therapies required (mostly repeated) HFH and had ~5-fold higher all-cause mortality over a long-term follow-up with median of 5.6 years after discharge upon index hospitalization for STEMI; (ii) CRP concentration during index hospitalization for STEMI and at one month after discharge were significantly higher in patients requiring HFH compared to those with no HFH; (iii) CRP_DC_ at discharge upon index hospitalization for STEMI significantly improved prediction of HFH; (iv) CRP_1M_ ≥ 2 mg/L at one month after discharge upon index hospitalization for STEMI, which indicates an ongoing low-grade systemic inflammation, was significantly more frequent in patients with HFH long-term than without (~58% of patients vs. ~43%); (v) CRP_AD_ at admission for index STEMI had no prognostic value for the risk of HFH over a long-term follow-up.

CRP concentration reflects inflammation that is implicated in post-infarct myocardial injury and repair as well as other processes triggered by acute MI such as accelerated atherosclerosis and endothelial dysfunction, all of which can be involved in the pathophysiology of HF [9,13,21,22,27]. A number of experimental data and human biomarker studies indicate the role of inflammation in enhancing infarct size, progression of post-infarct LV dysfunction and LVR, as well as in the development and outcome of chronic HF [5,10,11,12,13,21,25,27]. CRP level was shown to predict adverse long-term clinical outcome and diminished cardiorespiratory fitness in patients with symptomatic chronic ischemic HF, independently of other predictors such as BNP [25,41]. A few studies noted a relationship between CRP concentration during acute MI and post-infarct HF but were subject to limitations such as heterogenous populations with different types of acute coronary syndromes including STEMI patients and a lack of multiple serial high-sensitivity CRP measurements at various time-points of acute MI, a lack of guideline-based therapies in all patients, no use of primary PCI, no echocardiographic monitoring of LV function and LVR, no measurements of other valuable biomarkers such as BNP and/or a lack of long-term follow-up [18,19,20,39,40,46]. In addition, in previous studies addressing the prognostic value of CRP which were conducted exclusively in STEMI patients, HF or HFH over a long-term follow-up was not used as a single primary clinical endpoint [12,18,19,35,36,37,38,39,40,41]. A few studies which included HF post-STEMI as a clinical endpoint, had several limitations such as the use of composite endpoints, CRP measurement only at hospital admission for STEMI, lack of data on echocardiographic outcomes, no measurements of other biomarkers like BNP, and the use of guideline-based therapies only in portion of enrolled patients [18,19,39]. The present study is based on the protocol that includes several distinctive features compared with the previous trials. First of all, our study targets a homogenous population of patients with a first STEMI undergoing guideline-based therapies. In addition, while excluding confounding factors that could affect myocardial damage and LV function, our protocol includes serial multiple measurements of high-sensitivity CRP, multi-year follow-up, monitoring MACE, and HFH as a primary endpoint representing the validated symptomatic HF. The findings of this study raise important questions about inflammation-related mechanisms which can contribute to the initiation and progression of HF post-STEMI.

In our study, patients who developed symptomatic HF post-STEMI had large infarct size, severe LV dysfunction and LVR, and high CRP concentrations during the acute phase of STEMI, which indicate enhanced inflammation in infarcted area. Large infarct size, anterior location of STEMI, and LVSD were previously shown to be the predictors for readmissions for HF and a new-onset congestive HF, even in the era of primary PCI [1,5,7,39,47,48,49,50]. Also, LVR indicates an increased risk for HF post-MI [51]. Significant correlations between CRP, markers of myocardial necrosis, infarct size, and decreased LVEF were previously reported [5,10,12,36]. CRP was also associated with high LV end-diastolic pressure, BNP level, and post-infarct LV diastolic dysfunction and LVR [11,12,52,53,54].

Our findings of enhanced inflammation associated with enlarged infarcted area are consistent with several inflammation-related mechanisms triggered by STEMI which can lead to further myocardial injury, LV dysfunction, and LVR [5,10,11]. Thus, cardiac inflammation triggered by acute MI, even if treated with PCI, can promote a development of post-infarct HF [9,13,22,27]. Although reperfusion limits the myocardial necrotic area and inflammatory activation in STEMI, release of intracellular cytokines such as IL-1α and IL-33 contribute to local and systemic inflammatory responses. Activation of NLRP3 inflammasome in cardiomyocytes causes an inflammatory type of cell death that enhances infarct size and loss of functional myocardium [13,27]. The NLRP3 amplifies the inflammatory response by processing and secretion of IL-1β and IL-18. The role of IL-1 cytokines in inhibiting cardiac contractility is known from experimental studies in cells and animals. Activation of NLRP3 during acute MI in cells other than cardiomyocytes contributes indirectly to cardiac dysfunction. Release of radical oxygen species, enzymes, and cytokines in neutrophiles further injure the cardiomyocytes. Loss of endothelial cells function results in inappropriate vasodilation that further impairs coronary flow. In fibroblasts, IL-1β induces profibrotic changes contributing to enlargement of infarct scar. The link between IL-1 activity and HF is supported by elevated circulating levels of IL-1β as well as surrogate biomarkers such as IL-6 or CRP, each of which correlates with worsening HF symptoms and outcomes [23,24,25,26,27,45]. The NLRP3 expression was associated with adverse cardiac events including congestive HF in patients with acute coronary syndromes [55]. The reduction in CRP and IL-6 levels after administering IL-1β inhibitors confirms the findings of an enhanced IL-1 activity in patients with acute STEMI or post-infarct individuals with elevated CRP [56,57,58]. Also, a decrease in experimental ischemia-reperfusion-induced myocardial injury, apoptosis, infarct size, remodeling, and dysfunction were observed when the activity of IL-1α, NLRP3 inflammasome, and IL-1β were blocked [13,27].

Another important finding of our study is that patients requiring HFH over a long-term follow-up had persistent elevation of CRP_1M_ at one-month post-index hospitalization for STEMI (with a median ≥2 mg/L), which indicates ongoing low-grade inflammation. This chronic low-grade inflammation could result in escalation of atherosclerosis which may contribute to progression of HF. Importantly, in the HF+ group of patients with median CRP_1M_ ≥ 2 mg/L, the hospitalization for unstable angina leading to revascularization occurred in long-term follow-up twice as often as in the HF- group of patients with median CRP_1M_ < 2 mg/L, despite no differences between groups in the severity of coronary artery disease and management strategy. Low-grade inflammation was shown to be associated with an increased risk of atherosclerotic events, occurrence of HF, and worse outcome of patients with established HF [25,29,37,42,43,45]. In the CANTOS study of post-infarct patients with low-grade inflammation, individuals with CRP in the highest tertile were at 1.9 higher risk of HFH over a median period of 3.7 years compared to those with CRP in the lowest tertile [26]. In addition, anti-inflammatory strategies including IL-1 inhibition were effective in reducing long-term risk of atherosclerotic events and HFH in post-infarct patients with low-grade inflammation [26,57,59,60]. This benefit was independent of any effect on lipoproteins or blood pressure and was closely related with a decrease in inflammation as manifested by the greatest CRP reduction [57,61].

The findings of our study which indicate post-STEMI low-grade inflammation and its adverse effects are supported by preclinical data which suggested that acute inflammatory response following MI accelerates systemic atherosclerosis [14,62]. Large infarct size and consequently high inflammatory activation results in increased risk of systemic atherosclerosis exacerbation and recurrent atherosclerotic events [63]. Because chronic low-grade inflammation relates to ischemic events, the inflammation caused by acute MI may increase susceptibility to recurrent events and contribute to HF development. Activation of NLRP3 inflammasome and IL-1 cytokines such as IL-1β and IL-18 were shown to be critically involved in the ensuing post-infarct systemic inflammation and progression of atherosclerosis through contributing to the initiation, formation, growth, and rupture of atherosclerotic plaques [13,14,27,62,63]. Expression of the NLRP3 inflammasome in atherosclerotic plaques and in peripheral blood monocytes in patients with acute coronary syndrome were associated with coronary atherosclerosis and adverse cardiac events [55]. Also, IL-6 likely contributes causally to atherothrombosis and is associated with atherosclerosis progression [32,33,58].

Importantly, ongoing cardiac and systemic inflammation post-infarct can trigger and/or enhance inflammation-related endothelial dysfunction which contributes to the pathophysiology of numerous cardiovascular conditions and HF [15,16,28]. Inflammatory activation of endothelial cells by cytokines such as tumor necrosis factor (TNF)-α, IL-1α or IL-1β results in the increased expression of selectins and adhesion molecules augmenting an adherence of monocytes, reduced circulation of endothelial progenitor cells, dysregulated production and bioavailability of myocardial nitric oxide, augmented oxidative stress, and increased production of procoagulant mediators, which all aggravate endothelial dysfunction and predispose to atherothrombosis [15,16,27,28]. Thus, the endothelial dysfunction exacerbates systemic inflammation and pro-fibrotic processes, and further impairs vasodilation, angiogenesis, and vasculogenesis, which are all implicated in HF pathophysiology. Decrease in endothelium-dependent vasodilation can lead to coronary rarefaction with reduced coronary flow reserve as well as increased vascular stiffness and impaired arterial distensibility, augmenting myocardial damage [64,65]. In HF patients with reduced LVEF, as was the case in our study, endothelial dysfunction can be promoted via neurohormonal activation, altered shear stress, a decrease in the production of nitric oxide, and increased oxidative stress [65]. Circulating cytokines, such as TNF-α and IL-6, are related to the degree of endothelial dysfunction in HF, which correlates with progressive deterioration in HF functional class [15,65]. Thus, identification of elevated CRP as a risk marker for endothelial dysfunction may be useful to link a systemic marker of inflammation to progression of atherosclerotic disease and HF.

In our study, the patients requiring HFH had higher BMI and greater prevalence of DM compared to patients without HFH. Metabolic disorders such as obesity and DM (diabesity) are known to be associated with low-grade chronic inflammation (metaflammation) and endothelial dysfunction, and were identified as risk factors for HF and HFH, also in post-infarct patients with elevated CRP [1,7,26,28,66,67]. Several factors could be implicated in the metabolic-associated LV dysfunction that contributes to HF, such as LV hypertrophy, fat infiltration into the myocardium evolving into fibrosis, LV diastolic dysfunction, and endothelial dysfunction [28,68,69].

Our findings provide additional insights with relevance to recent trials on the effectiveness of anti-inflammatory therapies for reducing risk of HF and cardiovascular events. Existing evidence indicates that NLRP3 inflammasome activation during acute MI with enhanced IL-1 activity and increase in IL-6 and CRP levels contributes to the risk of post-infarct HF and that IL-1 targeting therapy may prevent HF development. Results of VCUART trials with IL-1 blockade with anakinra in patients with acute STEMI showed significant decrease in the systemic inflammatory response and lower incidence of death or new-onset HF or of death and HFH in 12-month follow-up [56]. CANTOS study demonstrated that therapy with canakinumab, an IL-1β inhibitor, resulted in a significant decrease in CRP and reduced rate of HFH and the composite of HFH or HF-related mortality during a median follow-up of 3.7 years in post-infarct patients with CRP ≥ 2 mg/L regardless of LVEF [26,57]. Importantly, these benefits were observed in patients who achieved on-treatment CRP < 2 mg/L. In addition, CANTOS provided strong support that targeting IL-1β or lowering downstream mediators of IL-1 action (such as IL-6 and CRP) associates with improved cardiovascular outcomes [58,61]. Previous studies also showed improvement in LV systolic function with IL-1 blockade and associated improvement in cardiorespiratory fitness in patients with established HF [70,71]. Because the CANTOS indicated that, following intervention with IL-1β inhibitor, there remains substantial residual inflammatory risk related to both IL-18 and IL-6, therapies that simultaneously inhibit IL-1β and IL-18 (such as NLRP3 inflammasome inhibitors) as well as directly target IL-6 signaling would be required [72]. Importantly, the results of COLCOT trial indicated that anti-inflammatory strategies other than direct IL-1 inhibition are effective in reducing long-term risk of ischemic cardiovascular events in patients with recent MI [59].

The significance of our study stems from scientific insights and clinical implications for the management of STEMI patients. CRP can aid at early identification of patients who are at-risk of HF over a multi-year period post-STEMI and prevention of hospital readmissions for HF and HF-related mortality. CRP adds prognostic value to information provided by the markers of infarct size and LV dysfunction. Knowledge on prognostic factors that are specific to HF adds to our understanding of the pathophysiological background of this complex condition and points to a need for developing new targeted therapies [1,3,4,73,74]. Biomarkers representing various pathomechanisms along with concomitant clinical assessment offer a desirable strategy for improving risk stratification and clinical decision making for post-STEMI patient management [5,10,11,18,41,43,73,75]. Our findings indicate that incorporating CRP into a multi-marker approach enhances risk discrimination and aids in a choice of targeted therapies for patients with STEMI. Measurement of CRP, a downstream inflammatory biomarker, can identify STEMI patients who are most likely to benefit from anti-inflammatory strategies for preventing HF, and ultimately enhance the current approaches of management of STEMI. Our findings raise important questions about inflammation-related mechanisms associated with post-infarct HF. Our study can be viewed as a bridge between clinical results and relevant inflammation-related pathophysiological concepts based on basic background and molecular mechanisms. Our findings support a need for further basic and clinical research on underlying mechanisms and new approaches for targeting inflammation to reduce morbidity and mortality related to post-infarct HF. Measurement of upstream inflammatory biomarkers could provide more insights on the role of enhanced inflammation as a risk of post-infarct HF and deserves special attention in future studies.

This is a single-center study; however, in this study well-controlled enrollment criteria, guideline-based therapies, adequate sample size, prospective long-term follow-up, use of HFH as the primary clinical endpoint that validates HF diagnosis, and collection of comprehensive set of biomarkers as well as clinical and echocardiographic data were utilized. Whereas the rigorous eligibility criteria for our study allowed us to avoid confounding factors and thus can be viewed as a strength of our study, on the other hand these criteria led to inclusion of only those patients who had a first STEMI and were subject to rapid primary PCI with successful reperfusion. This can have implications on our results, showing a well-preserved LV systolic function and relatively low incidence of post-infarct HFH. Potential circadian and seasonal variations in CRP concentration or genetic variants of CRP contributing to variability in CRP concentration were not accounted for. This is, however, a common limitation difficult to avoid in practice.

## 4. Materials and Methods

### 4.1. Study Design and Participants

We performed a prospective observational cohort study that included a homogenous population of patients who were hospitalized in our center for primary PCI due to a first STEMI and satisfied inclusion criteria. Details on the trial protocol and analyses related to the usefulness of CRP for the prediction of post-infarct LVSD and LVR were presented previously [5,10,11,75]. The present study extends the previous analyses and was designed to address prognostic value of CRP measured during index hospitalization for STEMI and at one month after discharge for the occurrence of HF over a long-term multi-year follow-up defined as a hospital readmission for HF in the whole study population regardless of LV function. Specifically, in terms of clinical endpoints we focused on the occurrence of validated HFH and all-cause mortality, which were prospectively captured in the long-term follow-up.

The study was performed in the Nicolaus Copernicus University in Toruń, Collegium Medicum in Bydgoszcz, Poland in accordance with the Declaration of Helsinki. Approval from the Bioethics Committee of the Collegium Medicum was obtained (KB 440/2004). All patients provided informed consent.

Inclusion criteria in the study protocol were: (1) first STEMI, (2) typical stenocardial chest pain of ≥30 min, (3) symptoms < 12 h before admission, (4) ST-segment elevation of ≥0.2 mV in V1 through V3 precordial leads or ≥0.1 mV in ≥ 2 other leads in ECG. Exclusion criteria included: age <18 and >80 years, female patients currently pregnant or of childbearing age who were not using contraception, prior MI, coronary revascularization or thrombolysis, cardiogenic shock on admission, HF symptoms of class ≥ III according to the New York Heart Association (NYHA) functional classification, creatinine concentration >176.8 µmol/L, hemodynamically significant valvular heart disease, primary cardiomyopathies, uncontrolled high blood pressure, atrial fibrillation, active inflammatory or neoplastic process on admission, and therapy with steroids, immunosuppressive agents, and non-steroidal anti-inflammatory drugs. The presence of the conditions, which were the exclusion criteria, was evaluated before enrollment of patient to the study by careful standard clinical evaluation by the attending cardiologist to exclude potentially confounding factors that could affect cardiac injury, cardiac function assessment, and inflammatory response.

The eligible consecutive patients were enrolled in this study at the Department of Cardiology and Internal Medicine, the University Hospital No. 1 in Bydgoszcz, Poland from December 2005 to December 2008. The incidence of total MACE, defined as the composite of all-cause death, non-fatal MI, ischemic stroke, hospitalization for unstable angina leading to revascularization, and HFH, was assessed in long-term follow-up post-index hospitalization for acute STEMI. The incidence of individual MACE components was also evaluated. Data on MACE in long-term follow-up (occurrence and dates of specific events) were acquired at in-person visits with study staff at 1 month, 6 months, and 12 months (based on clinical evaluation and hospital discharge cards collected from patients) after discharge upon index hospitalization for STEMI as well as from National Health Fund registry (based on the record of hospitalizations and data on death). Specifically, only the hospitalizations encoded as hospitalizations for MI, ischemic stroke, unstable angina leading to revascularization, and HFH were considered as specific MACE components. Non-fatal MI was defined using the universal definition of MI [76].

NYHA classification was used to evaluate the severity of HF prior to STEMI or HF at discharge upon index hospitalization for STEMI. Specifically, HF symptoms of ≥ III NYHA class with the objective evidence of abnormal heart structure or function at rest prior to STEMI was an exclusion criterion in this study. HF at discharge upon index hospitalization for STEMI was defined as the presence of HF symptoms corresponding to ≥ II NYHA class, i.e., shortness of breath at rest or during exertion and/or fatigue and signs of fluid retention such as pulmonary congestion or ankle swelling.

### 4.2. Study Endpoints

Hospitalization for HF (referred to as HFH) in long-term follow-up was the primary study endpoint. HFH required documented clinical and/or radiologic evidence of clinical HF and congestion. Specifically, HFH was defined as readmission post-index hospitalization for STEMI due to new or increasing symptoms and signs of HF including fluid retention or other objective evidence of HF, such as increasing dyspnea by one or more NYHA class(es), peripheral edema, bilateral post-tussive rales in at least lower third of lung fields, or ventricular gallop rhythm, in combination with a change in treatment to improve HF including parenteral use of diuretic [7]. The incidence of HFH in long-term follow-up after discharge upon index hospitalization for STEMI, time period between the discharge upon index hospitalization for STEMI and first HFH—as well as a number of repeated HFH post-index hospitalization for STEMI—were analyzed.

All-cause death in long-term follow-up after discharge upon index hospitalization for STEMI and the incidence of CRP_1M_ ≥ 2 mg/L at one month after discharge upon index hospitalization for STEMI were considered as secondary study endpoints. CRP_1M_ ≥ 2 mg/L was defined as a marker of low-grade systemic inflammation [29,57].

Comparisons of various characteristics including baseline clinical data, angiographic and echocardiographic parameters, biomarkers, and clinical outcomes between subgroups of patients requiring (HF+ group) or not requiring (HF- group) HFH in long-term follow-up were made.

Data on HFH were acquired at follow-up visits (based on hospital discharge cards collected from patients) and from National Health Fund registry based on the record of HF hospitalizations, specifically the hospitalizations encoded as HFH. Data on all-cause death in long-term follow-up were acquired from the National Health Fund registry.

### 4.3. Therapy

All patients received standard treatment following current guidelines on management of acute STEMI [77]. Immediately after diagnosis of STEMI, all patients received oral loading doses of clopidogrel (600 mg) and aspirin (300 mg), and intravenously unfractionated heparin (70 IU/kg, up to 5000 IU). Upon arrival to the hospital, in the cardiac catheterization laboratory, second dose of unfractionated heparin was administered intraarterially in a weight-adjusted manner (up to 100 IU/kg) or under activated clotting time guidance (to the target range of 200–250 s) when abciximab was intended. Coronary angiography and primary PCI were performed according to standard techniques using the femoral approach. The operator was blinded to the study protocol. Coronary stenting of culprit coronary vessel lesion was the technique of choice for all admitted patients. The use of abciximab and aspiration thrombectomy were done at the operator’s discretion. Thrombolysis in Myocardial Infarction (TIMI) 3 flow and TIMI Myocardial Perfusion Grade (TMPG) 3 were used to define reperfusion success. The analyses of all angiographic data were performed offline by two observers blinded to echocardiographic data, clinical outcomes, and biomarker levels.

Clopidogrel up to 1 year and aspirin, each at 75 mg q.d., were continued in all patients except for patients with allergy to aspirin. Concomitant medications included perindopril and long-acting metoprolol in doses adjusted for resting heart rate and blood pressure in all patients who tolerate these medications and without contraindications, regardless of blood pressure or LV function, and simvastatin at initial dose of 40 mg q.d. regardless of cholesterol level to achieve low-density lipoprotein (LDL) cholesterol < 80 mg/dL in all patients in the absence of contraindications. This treatment was administered in the acute phase of STEMI and was continued long term after STEMI. We monitored that these medications were continued throughout the study period for at least 12 months after hospital discharge post-STEMI and made recommendation for continued guideline-based pharmacotherapy beyond this 12-month period [78]. Other medications—such as spironolactone or non-potassium sparing diuretics—were administered depending on the indications.

### 4.4. Echocardiography

Two-dimensional transthoracic echocardiography employing the Doppler technique (SONOS 7500 Ultrasound System, Philips, Bothell, WA, USA) was performed at discharge upon index STEMI and six months after discharge following the American Society of Echocardiography recommendations [79]. Echocardiographic recordings were assessed by two experts blinded to the time point, clinical outcomes, and biomarker levels. Measurements from three consecutive cardiac cycles were averaged. LV systolic and diastolic function was evaluated. LV volumes and LVEF were calculated using the biplane method of discs (modified Simpson’s rule). LVR was defined as a relative > 20% increase in end-diastolic LV volume at six-month (LVR_6M_) follow-up compared with the baseline at discharge upon index STEMI [51]. Global LVSD was defined as the presence of LVEF ≤ 40% and was assessed at hospital discharge upon index hospitalization for STEMI (LVSD_DC_) and six months after index STEMI (LVSD_6M_) [77,79,80]. Regional LVSD and longitudinal LVSD were defined as the wall motion score index (WMSI) ≥ 1.7 and an average peak systolic mitral annular velocity (S’) by tissue Doppler imaging (TDI) ≤ 6.0 cm/s, respectively. LV diastolic dysfunction was defined as the presence of ratio of E/E’ ≥ 10, where E is early transmitral flow velocity (E) by pulsed wave Doppler, and E’ is diastolic mitral annular velocity by TDI. The inter- and intra-observer coefficients of variation (CV) for echocardiographic markers of both LV function and LVR assessed in first 50 patients were below 5.0% and below 2.5%, respectively.

### 4.5. Blood Sampling and Biomarkers

Peripheral venous blood samples were collected using ethylenediaminetetraacetic acid tubes. After centrifugation, the plasma was stored at −80 °C until analysis. High-sensitivity CRP plasma concentration was measured with an ultra-sensitive latex immunoassay (CRP Vario test, analyzer ARCHITECT ci 8200, Abbott Diagnostics, Abbott Park, IL, USA) at admission (CRP_AD_), 24 h after admission (CRP_24_), at discharge (CRP_DC_), and one month after discharge (CRP_1M_) upon an index hospitalization for STEMI. BNP plasma concentration was used to assess neurohormonal activation and hemodynamic stress. BNP concentration was measured with a chemiluminescent microparticle immunoassay (ARCHITECT ci 8200, Abbott Diagnostics, Abbott Park, IL, USA) at admission and discharge upon an index STEMI. Detection limit for CRP was 0.1 mg/L and for BNP 10 pg/L. Intra-assay CV was <2% for CRP and <5% for BNP, and for inter-assay <1% and <5%, respectively. Infarct size was estimated on the basis of the maximum creatinine kinase myocardial band (CK-MB) level, which was determined by direct chemiluminescent method at admission for STEMI and every 6 h starting immediately after primary PCI until 48 h.

### 4.6. Statistical Analysis

Most investigated variables did not obey normal distribution (based on Shapiro–Wilk test results). Statistics of continuous variables are reported as median values and interquartile ranges. Depending on the presence or absence of normal distribution, inter-group comparisons were performed with Student’s *t*-test for independent samples or Mann–Whitney unpaired rank sum test. Intra-group comparisons were made with Student’s *t*-test for paired samples or Wilcoxon matched-paired rank sum test. Categorical variables were compared using the χ2 test with the Yates correction. For this analysis, to assess associations between baseline concentrations of CRP and the risk of HFH during the trial follow-up period, CRP concentrations at four time points during and after index hospitalization for STEMI (admission, 24 h, discharge, and one month) were divided into increasing quartiles and the occurrence of future HFH was evaluated in each quartile of CRP. Univariate and multivariable logistic regression models were used to identify predictors of HFH in long-term follow-up. The first basic multivariable model was based on Generalized Linear/Nonlinear Model (GLZ). Given that the strong prognostic significance of depressed LVEF has long been proven, we expected that this first basic model may not reveal the potential prognostic value of other factors, especially high-sensitivity CRP during index STEMI. Therefore, a second multivariable model was examined to investigate if other markers including high-sensitivity CRP provide an added predictive value for HFH. In this second model, LVEF at hospital discharge upon index hospitalization for STEMI was purposely excluded. Relations between investigated variables and likelihood of HFH were estimated with odds ratios and 95% confidence intervals. Kaplan–Meier method was used to estimate probability of event-free survival. A two-sided *p* value of < 0.05 was considered significant. Statistical analysis was made using Statistica software system version 13 (TIBCO Software, Inc., Palo Alto, CA, USA).

In support of the presented analysis of CRP as a risk marker for HF over a multi-year period post-STEMI, we performed a post-hoc power analysis which indicates that our study has a sufficient statistical power to detect significant differences in CRP_DC_ between patients with and without HFH in long-term follow-up post-index hospitalization for STEMI. Specifically, using the GPower software (v.3.1.9.7) [81], we calculated that the total sample size of 204 patients consisting of 24 patients from HF+ group and 180 patients from HF- group provides 93% power at the 0.05 significance level to detect an effect size of 0.75 for the CRP_DC_ difference between HF+ and HF- groups which was observed in our study.

## 5. Conclusions

Persistent elevation in CRP concentration post-STEMI can serve as a risk marker and aid in identifying patients at increased risk of HF and HF-related mortality in multi-year period. Elevated CRP indicates ongoing cardiac and low-grade systemic inflammation which contributes to the development of post-infarct HF despite guideline-based therapies. Elevated CRP_DC_ at discharge upon index hospitalization for STEMI significantly improved prediction of hospitalization for HF in multi-year follow-up. In addition, patients with CRP_1M_ ≥ 2 mg/L at one month after index hospitalization for STEMI exhibited higher risk of HF hospitalization long-term. Measurement of CRP during hospitalization for STEMI and at one month after discharge can aid in identifying patients who are most likely to benefit from early implementation of anti-inflammatory strategies for preventing HF in long-term follow-up. The key role of inflammatory activation in the pathophysiology of post-infarct HF supports a need for further basic and clinical research on underlying mechanisms and new approaches for targeting inflammation to reduce morbidity and mortality related to post-infarct HF.

## Figures and Tables

**Table 1:** Baseline characteristics of patients during index hospitalization for ST-elevation myocardial infarction who were or were not hospitalized for heart failure in long-term follow-up.

Variable	Patients with HF Hospitalization (*n* = 24)	Patients without HF Hospitalization (*n* = 180)	*p*-Value between Patients with or without HF Hospitalization
Age (years)	57.5 (53.0–66.5)	56.0 (50.0–64.0)	0.19
Gender (male: female) *n* (%)	17:7 (71.0: 29.0)	139:41 (77.0:23.0)	0.488
Body mass index (kg/m^2^)	29.4 (26.8–30.8)	26.2 (24.1–29.0)	0.048
Hypertension *n* (%)	11 (45.8)	73 (40.6)	0.622
Diabetes mellitus *n* (%)	9 (37.5)	28 (15.6)	0.009
STEMI pain duration (min)	251.5 (150.5–395.5)	195.0 (140.5–286.0)	0.047
Anterior STEMI *n* (%)	20 (83.3)	69 (38.3)	<0.001
HF prior to STEMI (≤II NYHA class) *n* (%)	1 (4.2)	6 (3.3)	0.175
HF at discharge upon STEMI (≥II NYHA class) *n* (%)	8 (33.3)	12 (6.7)	<0.001
**Treatment at discharge upon index hospitalization for STEMI**			
Long-acting metoprolol *n* (%)	24 (100.0)	178 (98.9)	0.356
Perindopril *n* (%)	24 (100.0)	176 (97.8)	0.714
Simvastatin *n* (%)	24 (100.0)	179 (99.4)	0.928
Spironolactone *n* (%)	5 (20.8)	12 (6.7)	0.02
Non-potassium sparing diuretics *n* (%)	6 (25.0)	7 (3.9)	<0.001
**Biochemical parameters during index hospitalization for STEMI**			
Creatinine at admission (µmol/L)	88.4 (79.6–98.1)	79.6 (71.6–92.8)	0.113
Glucose at admission (mmol/L)	8.1 (6.8–11.2)	7.5 (6.7–9.2)	0.248
Glycated hemoglobin at admission (%)	6.2 (5.6–7.3)	6.0 (5.6–6.6)	0.38
Leukocyte count at admission (10^3^/µL)	11.4 (10.2–13.3)	11.2 (9.1–13.2)	0.557
Leukocyte count 24 h after admission (10^3^/µL)	11.1 (9.2–13.5)	10.1 (8.5–11.6)	0.057
LDL cholesterol at admission (mmol/L)	3.5 (3.0–4.6)	3.8 (3.3–4.5)	0.474
CK-MB at admission (U/L)	32.5 (20.0–82.0)	25.5 (18.0–41.5)	0.01
CK-MB_max_ (U/L)	174.0 (59.0–225.0)	96.0 (55.5–145.0)	0.001
TnI_max_ (ng/mL)	>50.0 (18.5- > 50.0)	35.1 (10.1- > 50.0)	<0.001
CRP at admission (mg/L)	2.37 (1.29–3.82)	1.68 (0.93–3.27)	0.075
CRP at 24 h (mg/L)	24.12 (12.69–33.33)	9.34 (5.26–16.29)	<0.001
CRP at discharge (mg/L)	19.94 (12.36–35.74)	9.21 (4.46–16.45)	<0.001
CRP at one month (mg/L)	2.57 (1.46–4.98)	1.54 (0.79–3.04)	0.01
BNP at admission (pg/mL)	74.6 (29.1–156.9)	50.9 (25.5–89.9)	0.001
BNP at discharge (pg/mL)	336.5 (227.2–717.5)	106.7 (62.2–169.2)	<0.001
**Angiographic indices during primary PCI for index STEMI**			
LAD/non-LAD *n* (%)	20 (83.3) /4 (16.7)	73 (40.6) /27 (59.4)	<0.001
TIMI 3 flow pre-PCI *n* (%)	6 (25.0)	52 (28.9)	0.601
TIMI 3 flow post-PCI *n* (%)	23 (95.8)	167 (92.8)	0.028
TMPG 3 post-PCI *n* (%)	10 (41.7)	84 (46.7)	0.644
Stent use *n* (%)	24 (100.0)	178 (98.9)	0.249
Multivessel disease *n* (%)	18 (75.0)	105 (58.3)	0.117
Abciximab use *n* (%)	11 (45.8)	39 (21.7)	0.011

Data represent median values with corresponding interquartile range (in parenthesis). Abbreviations: BNP—B-type natriuretic peptide; CK-MB_max_—maximum activity of creatine kinase-MB; CRP—high-sensitivity C-reactive protein; HF—heart failure; LAD—infarct-related left descending artery; LDL cholesterol—low-density lipoprotein cholesterol; NYHA—New York Heart Association; PCI—primary percutaneous coronary intervention; STEMI—ST-elevation myocardial infarction; TIMI—Thrombolysis in Myocardial Infarction score; TMPG—TIMI Myocardial Perfusion Grade; TnI_max_—maximum concentration of troponin I.

**Table 2:** Echocardiographic characteristics of patients with acute ST-elevation myocardial infarction at one and six months after discharge upon index hospitalization for STEMI who were or were not hospitalized for heart failure in long-term follow-up.

Variable	Patients with HF Hospitalization (*n* = 24)	Patients without HF Hospitalization (*n* = 180)	*p*-Value between Patients with or without HF Hospitalization
**At discharge upon index hospitalization for STEMI**			
LVEDVI (mL/m^2^)	51.3 (42.8–62.2)	49.1 (43.7–59.6)	<0.001
LVESVI (mL/m^2^)	29.7 (23.5–37.8)	26.4 (23.0–33.0)	<0.001
LVMI (g/m^2^)	140.2 (103.2–144.9)	110.8 (93.1–129.0)	<0.001
LVEF (%)	38.5 (36.5–47.3)	45.6 (44.0–50.3)	<0.001
WMSI (pts)	1.81 (1.44–1.84)	1.44 (1.38–1.69)	<0.001
S’ (cm/s)	6.0 (5.1–6.8)	7.2 (6.3–8.4)	<0.001
DT (ms)	147.5 (140.0–177.5)	160.0 (145.0–190.0)	0.031
E/E’ (-)	12.3 (9.4–17.2)	10.2 (8.2–12.0)	0.01
**At six months after discharge upon index hospitalization for STEMI**			
LVEDVI (m-L/m^2^)	77.6 (69.4–83.0)	55.2 (47.7–66.1)	<0.001
LVESVI (mL/m^2^)	46.4 (40.6–56.6)	28.1 (24.3–34.5)	<0.001
LVMI (g/m^2^)	141.8 (130.4–162.5)	112.1 (101.5–129.2)	<0.001
LVEF (%)	37.0 (36.4–50.0)	47.0 (43.0–52.3)	<0.001
WMSI (pts)	1.88 (1.38–1.88)	1.44 (1.31–1.63)	<0.001
S’ (cm/s)	5.7 (5.0–6.8)	7.1 (6.3–8.2)	<0.001
DT (ms)	160.0 (145.0–185.0)	175.0 (155.0–200.0)	0.017
E/E’ (-)	13.5 (11.1–16.1)	9.2 (7.8–10.9)	<0.001

Data represent median values with corresponding interquartile range (in parenthesis). Abbreviations: DT—deceleration time of early transmitral flow; E—peak velocity of early transmitral flow; E’—average peak early diastolic mitral annular velocity; LVEDVI-left ventricular end-diastolic volume index; LVEF—left ventricular ejection fraction; LVESVI-left ventricular end-systolic volume index; S’—average peak systolic mitral annular velocity; STEMI—ST-elevation myocardial infarction; WMSI—wall motion score index.

**Table 3:** Predictors of hospitalization for heart failure in long-term follow-up post index hospitalization for ST-elevation myocardial infarction based on univariate and multivariable analyses.

Variable	OR	95% CI	*p*-Value
Univariate Analysis			
WMSI at discharge (for a one-point increase)	88.98	14.706–538.333	<0.00001
Body mass index (for a 10 kg/m^2^ increase)	3.54	1.348–9.301	0.001
Heart failure at discharge upon index STEMI	2.24	1.500–3.335	<0.0001
STEMI pain duration (for a 1 min increase of logarithm of time)	2.02	1.020–4.002	0.044
Glycated hemoglobin (for a 1% increase)	1.37	1.044–1.794	0.023
WBC 24 h after admission (for a 10^3^/µL increase)	1.15	1.011–1.310	0.033
E/E’ at discharge (for a one-point increase)	1.13	1.046–1.225	0.002
Glucose at admission (for a 1 mmol/L increase)	1.1	1.041–1.171	0.001
LVESVI at discharge (for a 1 mL/m^2^ increase)	1.08	1.055–1.101	<0.00001
CK-MB_max_ (for a 10 U/L increase)	1.06	1.023–1.096	0.0003
TnI_max_ (for a 1 ng/mL increase)	1.05	1.013–1.082	0.006
CRP at 24 h after admission (for a 10 mg/L increase)	1.03	1.016–1.038	<0.0001
CRP at discharge (for a 10 mg/L increase)	1.03	1.017–1.051	0.0001
BNP at discharge (for a 100 pg/mL increase)	1.02	1.001–1.003	<0.00001
CK-MB at admission (for a 10 U/L increase)	1.01	1.004–1.011	0.00011
BNP at admission (for a 100 pg/mL increase)	1.01	1.002–1.008	0.0001
LVEF at discharge (for a 1% increase)	0.87	0.827–0.919	<0.00001
S’ at discharge (for a 1 cm/s increase)	0.42	0.291–0.592	<0.00001
**Multivariable Analysis**			
CK-MB at admission (for a 10 U/L increase)	1.06	1.010–1.109	0.017
LVEF at discharge (for a 1% increase)	0.92	0.869–0.981	0.01
S’ at discharge (for a 1 cm/s increase)	0.56	0.375–0.827	0.004
**Multivariable Analysis with LVEF at Discharge Upon Index Hospitalization for STEMI Excluded**			
WMSI at discharge (for a one-point increase)	105.37	14.936–743.453	0.000003
Body mass index (for a 10 kg/m^2^ increase)	3.01	1.003–9.025	0.049
STEMI pain duration (for a 1 min increase of logarithm of time)	2.11	1.128–3.994	0.019
CRP at discharge (for a 10 mg/L increase)	1.02	1.002–1.040	0.031

Results are presented according to decreasing values of odds ratios. Abbreviations: BNP—B-type natriuretic peptide; CI—confidence interval; CK-MB—activity of isoenzyme MB of creatine kinase; CK-MB_max_—maximum activity of isoenzyme MB of creatine kinase; CRP—high-sensitivity C-reactive protein; E—peak velocity of early transmitral flow; E’—average peak early diastolic mitral annular velocity; LVEF—left ventricular ejection fraction; LVESVI—left ventricular end-systolic volume index; OR—odds ratio; S’—average peak systolic mitral annular velocity; STEMI—ST-elevation myocardial infarction; TnI_max_—maximum concentration of troponin I; WBC—leukocyte count; WMSI—wall motion score index.

## Data Availability

The data presented in this study are available on request from the corresponding author. The data are not publicly available due to privacy restrictions.

## Abbreviations

BNP	B-type natriuretic peptide
CK-MB	Creatinine kinase myocardial band
CRP	C-reactive protein
CRP_AD_	C-reactive protein concentration at hospital admission
CRP_24_	C-reactive protein concentration 24 h after hospital admission
CRP_DC_	C-reactive protein concentration at hospital discharge
CRP_1M_	C-reactive protein concentration one month after hospital discharge
HF	Heart failure
HFH	Hospitalization for heart failure
LVEF	Left ventricular ejection fraction
LVSD	Left ventricular systolic dysfunction
LVSD_DC_	Left ventricular systolic dysfunction at hospital discharge
LVSD_6M_	Left ventricular systolic dysfunction six months after hospital discharge
MI	Myocardial infarction
PCI	Percutaneous coronary intervention
STEMI	ST-elevation myocardial infarction
WMSI	Wall motion score index
